# A Protocol for Nurse-Practitioner Led Cardiovascular Follow-Up After Pregnancy Complications in a Socioeconomically Disadvantaged Population

**DOI:** 10.3389/fcvm.2019.00184

**Published:** 2020-01-08

**Authors:** Emily Aldridge, Petra E. Verburg, Susan Sierp, Prabha Andraweera, Gustaaf A. Dekker, Claire T. Roberts, Margaret A. Arstall

**Affiliations:** ^1^Adelaide Medical School, Robinson Research Institute, University of Adelaide, Adelaide, SA, Australia; ^2^Faculty of Health and Medical Sciences, Adelaide Medical School, University of Adelaide, Adelaide, SA, Australia; ^3^Department of Cardiology, Lyell McEwin Hospital, Adelaide, SA, Australia; ^4^Department of Obstetrics and Gynaecology, Rijnstate Hospital, Arnhem, Netherlands; ^5^Department of Obstetrics and Gynaecology, Lyell McEwin Hospital, Adelaide, SA, Australia

**Keywords:** cardiovascular disease, pregnancy complications, postpartum follow-up, lifestyle intervention, prevention, women

## Abstract

**Background:** Women who experience pregnancy complications have an increased risk of future cardiovascular disease when compared to their healthy counterparts. Despite recommendations, there is no standardized cardiovascular follow-up in the postpartum period for these women, and the Australian follow-up protocols that have been previously described are research-based. This study proposes a new model of care for a nurse practitioner-led postpartum intervention clinic for women who experience severe hypertensive disorders of pregnancy, gestational diabetes mellitus requiring medication, severe intrauterine growth restriction, idiopathic preterm delivery, or placental abruption, in a socioeconomically disadvantaged population.

**Methods:** All women receiving antenatal care or who deliver at the Lyell McEwin Hospital, a tertiary acute care facility located in the northern Adelaide metropolitan area, following a severe complication of pregnancy are referred to the intervention clinic for review at 6 months postpartum. A comprehensive assessment is conducted from demographics, medical history, diet and exercise habits, psychosocial information, health literacy, pathology results, and physical measurements. Subsequently, patient-specific education and clinical counseling are provided by a specialized nurse practitioner. Clinic appointments are repeated at 18 months and 5 years postpartum. All data is also collated into a registry, which aims to assess the efficacy of the intervention at improving modifiable cardiovascular risk factors and reducing cardiovascular risk.

**Discussion:** There is limited information on the efficacy of postpartum intervention clinics in reducing cardiovascular risk in women who have experienced pregnancy complications. Analyses of the data collected in the registry will provide essential information about how best to reduce cardiovascular risk in women in socioeconomically disadvantaged and disease-burdened populations.

## Introduction

As well as a major cause of death, cardiovascular disease (CVD) is a significant cause of years of life lost for Australian women, resulting in 87,323 years life lost in 2015 (1). CVD remains a national public health crisis, costing over $5 billion in 2012–13 in healthcare for admitted patients (both male and female) and accounting for over 11% of total health expenditure (2). Awareness of CVD risk among women remains poor, despite them being almost three times more likely to die from CVD than from breast cancer (3). Emerging evidence demonstrates that although both men and women presenting with acute myocardial infarction experience the same symptoms (4), sex disparities in revascularization procedures still exist; a recent Australian cohort study reported that even after adjusting for age, sociodemographic and health-related variables, men were 50% more likely than women to receive percutaneous coronary intervention (PCI) or coronary artery bypass grafting (CABG) after admission to hospital with acute myocardial infarction (adjusted HR = 1.51, 1.38–1.67) (5). Men presenting with angina were also 150% more likely to receive PCI or CABG than women presenting with angina (adjusted HR = 2.44, 2.16–2.75) (5). Furthermore, both local and international literature has demonstrated that women who present with premature heart disease (age ≤ 55 years) take longer to receive emergency intervention and are more likely to experience worse outcomes following a cardiovascular event than men (6–8). Women who are socioeconomically disadvantaged are at even higher risk (9), and women in the lowest socioeconomic group in Australia have up to a 39% increase in number of years of life lost from CVD when compared to the national average (1, 10).

There now exists a plethora of literature detailing the relationship between pregnancy complications (including hypertensive disorders of pregnancy, gestational diabetes mellitus, intrauterine growth restriction, idiopathic preterm birth, and placental abruption) and an increased risk of future CVD (11–19). During pregnancy, maternal physiology undergoes complex and substantial changes, resulting in heightened organ function to cope with fetal demand and to maintain pregnancy (20). Failure to adapt to this physiological “stress test” and subsequent development of a complication of pregnancy is associated with an increased risk for future CVD. Pregnancy can therefore be viewed as a “window” into a woman's future health (16); however, it remains unclear if pregnancy reveals a pre-existing, underlying susceptibility to future CVD, or if the pregnancy itself leads to pathological changes, including inflammation and endothelial dysfunction, which in turn leads to a higher risk of future CVD.

In Australia, ~25% of all pregnancies are complicated by at least one of the previously described pregnancy complications. The subsequent risk of heart disease following a complicated pregnancy is significant; for example, in a recent meta-analysis, preeclampsia was found to be independently associated with an increased risk of future heart failure (risk ratio [RR] = 4.19; 95% confidence interval [CI], 2.09–8.38), coronary heart disease (RR = 2.50; 95% CI, 1.43–4.37), CVD death (RR = 2.21; 95% CI, 1.83–2.66), and stroke (RR = 1.81; 95% CI, 1.29–2.55) (11). Women with a history of preeclampsia are also twice as likely to develop Type 2 diabetes mellitus (RR = 2.37; 95% CI, 1.89–2.97) (21), and three times more likely to develop hypertension than their healthy counterparts (RR = 3.13, 95% CI, 2.51–3.89) (22).

The onset of hypertension and metabolic disease following preeclampsia has been shown to occur as early as 2 years postpartum (23, 24). Similarly, metabolic disease can declare itself during pregnancy, manifesting as gestational diabetes mellitus, resolving clinically postpartum only to recur in subsequent years (19). However, there remains a lack of knowledge and awareness among the broader medical community of the need for ongoing care after preeclampsia and other pregnancy complications that are manifestations of maternal placental syndromes or metabolic disease, despite wide acceptance of the traditional risk factors for CVD.

Although there exists broad international and national guidelines for postpartum follow-up following hypertensive disorders of pregnancy and gestational diabetes (25–27), these are somewhat inconsistent and there remains little consensus on the ideal time to commence follow-up. Other pregnancy complications within the maternal placental syndrome spectrum that are associated with an increased risk of CVD, such as intrauterine growth restriction and preterm delivery, do not have any specific postpartum follow-up guidelines. Emergent literature has explored the feasibility and effectiveness of an early postpartum intervention for women who have experienced a pregnancy complication in an effort to reduce cardiovascular risk (16, 28–31). These interventions promote education regarding cardiovascular risk and offer advice, treatment and prevention strategies to improve both short- and long-term cardiovascular and metabolic health. Despite some positive initiatives with research-based approaches (32), there remains no standardized model of care for Australian women who experience pregnancy complications. Furthermore, there is a paucity of literature focusing on low socioeconomic, disease-burdened populations, where pregnancy complication rates are likely higher than the national average of 25%. Most follow-up protocols for women who experience pregnancy complications are currently conducted within the context of research and are not considered routine care clinics; thereby the possibility of bias exists.

This article describes a new postpartum intervention clinic and registry introduced as part of standard care, that commenced in late 2018 for women who experienced severe complications during pregnancy in a low socioeconomic population located in the northern suburban areas of Adelaide in South Australia. The primary aim of the associated registry is to facilitate the assessment of the effectiveness of the clinic as a primary prevention program for women with a history of complicated pregnancies.

## Materials and Methods

### Study Design and Setting

This postpartum intervention clinic is routine care for women who receive antenatal care at the Lyell McEwin Hospital (based in the northern metropolitan area of Adelaide, South Australia) and experience at least one serious complication of pregnancy.

The registry associated with this clinic includes all clinical, demographic and lifestyle data collected during the clinic appointments. All patients attending this clinic are included in the registry, and data is stored confidentially in an electronic database. The Central Adelaide Local Health Network Human Research Ethics Committee waived the requirement for written informed consent for participants in this study due to its primary aim as a quality control study for a standard care hospital outpatient clinic. This approval is in accordance with national legislation and institutional requirements.

The postpartum intervention clinic is led by a nurse practitioner who has both vast experience in cardiac rehabilitation practices and has completed further education in postpartum cardiovascular follow-up. A nurse practitioner was the preferred clinician of choice as they can provide holistic and well-rounded care. A nurse practitioner is a more cost-effective choice than a medical practitioner, as well as a more practical option than allied health professionals, who tend to specialize in only one specific area. The clinic is also supported by a research team that assists with the data collection process.

### Participants: Eligibility and Referral

Women of any parity are referred to the postpartum intervention clinic as routine practice by the antenatal care team either during pregnancy or during admission for delivery after being diagnosed with at least one pregnancy complication meeting the referral eligibility criteria (Table 1). There are currently no maternal age or parity restrictions for referral to the clinic. Non-English-speaking patients are also accepted, and interpreters are booked accordingly.

**Table 1:** Referral eligibility criteria for the postpartum pregnancy complication clinic.

Hypertensive disorders requiring any medication and/or delivery at <37 weeks' gestation
Gestational diabetes mellitus requiring any medication
Intrauterine growth restriction and/or delivering a small-for-gestational age neonate (<5th percentile)
Preterm delivery occurring at <34 weeks' gestation
Placental abruption

Due to the overwhelming number of patients experiencing a complication of pregnancy, the clinic is currently only able to accept patients at the most severe end of the disease spectrum, on the assumption that this group is at greatest risk of future CVD. There are some limited data supporting this assumption (33–35). Table 1 describes the classification and severity of each condition required for acceptance into the clinic.

Referral typically takes place during pregnancy or at time of admission for delivery. The obstetrics team considers referral to the clinic for all patients as a standard component of discharge from hospital to ensure all eligible women are offered an appointment.

### Study Procedure

Patients are scheduled an appointment in the clinic at 6 months postpartum. Clinic visits are conducted as outpatient appointments within the Department of Cardiology at the Lyell McEwin Hospital. Patients receive a letter advising of the appointment date and time. The letter also contains some basic information about the link between pregnancy complications and CVD to provide rationale for attending the appointment. Enclosed with the letter is a pathology request form for fasting blood and urine tests to be completed prior to clinic attendance. A further SMS reminder the day prior to their scheduled appointment is also sent.

Fasting blood and urine samples are collected by trained phlebotomists at SA Pathology ideally at least 1 day prior to the scheduled appointment. Any patients who fail to provide blood and urine specimens in advance are still able to attend their appointments, but they are asked to have their pathology tests completed within the week following the appointment to allow for a complete assessment and accurate advice. These patients are either rebooked for a second appointment to discuss their pathology results, or they receive their results and any additional counseling from the nurse practitioner via telephone.

Information obtained from patients during the appointment includes demographics, medical history, family history, substance use, breastfeeding history and status, diet and exercise habits, psychosocial information, health literacy, pathology test results, and physical measurements. Patients are firstly asked to complete several short questionnaires themselves, and the remainder of the data is obtained by a clinic researcher or the nurse practitioner. Information is originally recorded on paper copies and is later entered into the medical case notes. The information is also recorded electronically in the registry. Once collected, this data is considered by the nurse practitioner and the patient receives specific advice and clinical counseling to improve modifiable cardiovascular risk factors. A detailed account of the outcome measures is described below.

Patients are automatically scheduled follow-up appointments at 18 months and 5 years postpartum. Most measures and questionnaires are repeated at the follow-up appointments. Pathology tests are repeated at all 5 year appointments but are only repeated at 18 months where clinically indicated. Initial appointments typically last for 45–60 min. Review appointments last for ~30 min.

### Intervention

The intervention within the postpartum clinic specifically refers to the education and clinical counseling provided by the nurse practitioner. Development of the style and content of the intervention was informed by the Maternal Health Clinic in Kingston, Canada (29). A face-to-face style intervention was chosen due to the need for obtaining measurements and performing physical assessments. There is also a significant lack of literature exploring the effectiveness of remote, online or telephone lifestyle interventions for women experiencing pregnancy complications. The decision regarding time of intervention at 6 months was somewhat influenced by the Canadian clinic (29), although there are suggested benefits of intervening prior to any subsequent pregnancies (36).

A major point of difference in the clinic described in this protocol is that it is led by a nurse practitioner. Nurse-led interventions are becoming increasingly popular in hospitals and other health services in Australia and worldwide (37). Nurse practitioners have expert knowledge of specific health conditions, which is ideal for leading clinics that rely on counseling and education. Although there remains a paucity of research demonstrating the specific effectiveness of nurse practitioner-led clinics, nurse-led clinics for instilling secondary prevention strategies in patients with CVD have demonstrated a positive impact on all-cause mortality rates, rates of significant cardiac events and medication adherence among patients (38). In Australia, nurse practitioners represent a more cost-effective solution for multidisciplinary education clinics than medical specialists, and provide a more holistic approach than allied health specialists who normally have expertise in one specific domain. Furthermore, nurses and nurse practitioners are generally able to undertake longer appointments, thereby establishing better rapport with the patient and obtaining assessments that are more comprehensive. Emergent literature explores these benefits and details the scope of specific health areas that may reap advantages from utilizing nurse-led interventions (37, 39, 40) but further research in this field is necessary.

Following collection of all relevant information, the nurse practitioner considers the patients' results and provides specific health and lifestyle education and clinical counseling based on improving modifiable cardiovascular risk factors. Breastfeeding practices are considered by the nurse practitioner during education and clinical counseling to ensure any recommended health practices are safe for mothers that are still breastfeeding.

The nurse practitioner provides general dietary advice based on the Australian Heart Foundation Heart Healthy Eating Patterns position statement which broadly recommends eating plenty of fruit, vegetables and whole grains, eating a variety of heart-healthy protein sources and limiting the intake of red meat, consuming healthy dairy choices (and choosing reduced-fat dairy if blood cholesterol is high), choosing healthy fat sources, and using herbs and spices in the place of salt (14). Advice regarding numbers of daily serves of each food group is based upon those recommended by the Australian Dietary Guidelines (41).

Physical activity advice is based on *Australia's Physical Activity and Sedentary Behavior Guidelines (18–65 years)* which recommend being active on most or all days, engaging in muscle strengthening exercises at least twice weekly, and accumulating 150–300 min of moderate-intensity exercise, or 75–150 min of vigorous-intensity, or a combination of these, each week (42). These guidelines are also recommended by the Australian Heart Foundation (43), although there are some minor differences.

Any abnormalities in the pathology results will prompt further investigation, and medications may be prescribed where necessary. Additional diagnostic tests, including echocardiograms, ambulatory blood pressure monitoring, and ultrasounds may also be ordered where required. The nurse practitioner will also further refer patients where appropriate to additional health providers, including general practitioners, mental health services, allied health or medical specialists. Formal reports summarizing each patient's results are forwarded to their nominated general practitioner.

### Outcome Measures

The following outcome measures will be recorded and compared at baseline (6 months postpartum), 18 months postpartum and 5 years postpartum. Cardiovascular risk scores will not be routinely calculated, as there are none suitably validated for Australian women under the age of 35 years.

#### Demographics

Basic details including social demographics (e.g., marital status, education, occupation, household income, etc.), medical history, family health history, substance use (e.g., smoking, alcohol, and other drugs), obstetric history and breastfeeding history are collected from a combination of patient self-reporting and medical case note review where necessary. This information will be updated at each appointment to ensure the most recent and accurate data is captured.

#### Physical Activity

The International Physical Activity Questionnaire (IPAQ) Long Form for English, which has been extensively tested for reliability and validity, is used to assess all occupational, incidental and planned physical activity (44). Data collected with this questionnaire will be reported as both continuous and categorical measure. The continuous measure is reported as median metabolic equivalent of task (MET-minutes) and calculates the median and interquartile range values using specific formulae for walking, moderate-intensity activities and vigorous-intensity activities across four domains (work, active transport, domestic and garden, and leisure time). The total volume of physical activity and the number of days and sessions are graded into one of three categories according to the scoring protocol; “low,” “moderate,” and “high.” This questionnaire is completed at all visits to assess any changes in physical activity habits.

#### Dietary Intake

An informal food frequency style questionnaire developed by our research group assesses adherence to major food groups and identifies general dietary intake compared against the Australian Dietary Guidelines 2013 (45). This tool is not validated, but provides detailed information of intake of whole grains, dairy, proteins, fruit and vegetables, discretionary foods, and drinks. This questionnaire identifies dietary deficiencies and highlights areas that may require improvement and is repeated at all appointments.

#### Psychosocial Measures

The General Anxiety Disorders (GAD 7-item scale) is a validated self-report questionnaire that assesses symptoms of anxiety over the past fortnight (46). Symptoms of depression over the past fortnight are assessed with the validated Patient Health Questionnaire (PHQ 9-item scale) (47). The GAD 7 and PHQ 9 are completed at all appointments to assess current symptoms of anxiety and depression.

The Medical Outcomes Study Social Support Scale (MOS-SSS) is a validated, frequency scale questionnaire that assesses the level of physical and emotional support available to the patient (48). This questionnaire is only completed by patients at the baseline visit to provide insight into the level of support available to new mothers.

#### Health Awareness

Health awareness is assessed via a mixed-methods survey to determine knowledge and awareness of pregnancy complications and the associated risk of future CVD. The survey questions and answer options are available in Table 2. This survey is also only completed at the first clinic appointment.

**Table 2:** Health awareness survey.

	**Question**	**Answer option**
1	Before receiving your clinic referral letter, were you aware that you had experienced a complication of pregnancy?	Yes/No
2	Which complication/s did you have during pregnancy?	*Select all that apply* Gestational hypertension Preeclampsia Eclampsia HELLP syndrome Gestational diabetes Growth-restricted baby Placental abruption Delivery of a small for gestational age baby Other (text line)
3	How satisfied are you with the care you received after being diagnosed with a pregnancy complication?	*Score out of 10, where 0 is very unsatisfied and 10 is very satisfied* 0–10
4	After diagnosis of your complication, were you made aware of the link between pregnancy complications and the higher risk of heart disease down the track?	*If no, skip to question 6* Yes/No
5	Who told you about this link?	*Select all that apply* Obstetrician Midwife/nurse General practitioner Cardiologist Other
6	Please tick all of the pregnancy complications that you have heard of:	*Select all that apply* Gestational hypertension Preeclampsia Eclampsia HELLP syndrome Gestational diabetes Growth-restricted baby Placental abruption Delivery of a small for gestational age baby
7	Where did you first hear about any one of these complications?	*Select all that apply* Obstetrician Midwife/nurse General practitioner Other healthcare provider Pregnancy book/magazine Family member/friend Website Social media Mobile app Television Other (text line)

#### Physical and Hemodynamic Measurements

The measurements obtained at the appointment clinic include waist circumference, weight and height, and are taken by trained clinic staff. Waist circumference is measured at the midpoint between the lowest rib and the topmost point of the iliac crest. Body mass index (BMI) is also calculated. Hemodynamic measurements include peripheral blood pressure, central blood pressure, pulse rate, and augmentation index, all performed on the USCOM BP+ [USCOM, Sydney, Australia]. Referral pregnancy booking weight (typically taken from 6 to 12 weeks' gestation in the antenatal clinic) is recorded from the medical case notes for comparison with weight at the time of the clinic appointment. Reliable pre-pregnancy weights and measures of weight gain in pregnancy are not available. All physical and hemodynamic measurements are conducted at each clinic appointment.

#### Cardiovascular Biomarkers

Fasting blood and urine samples are collected and assayed at SA Pathology prior to clinic attendance. The full panel of requested pathology tests are summarized in Table 3. These tests are repeated at 18 months postpartum only when deemed clinically necessary by the nurse practitioner but are all repeated at 5 years postpartum.

**Table 3:** Requested pathology tests for postpartum review at the postpartum pregnancy complication clinic.

**Pathology test**	**Unit of measurement**
*Urine chemistry*	
Creatinine	mmol/L
Albumin urine	mg/L
Albumin/creatinine ratio	mg/mmol
*Lipid studies*	
Total cholesterol	mmol/L
Triglyceride	mmol/L
HDL cholesterol	mmol/L
LDL cholesterol	mmol/L
Total cholesterol/HDL ratio	
Non-HDL cholesterol	mmol/L
*Glucose studies*	
HbA1c	mmol/L & %
Glucose	mmol/L
Insulin	mU/L
*Complete blood examination*	
Hemoglobin	g/L
White cell count	× 10^∧^9/L
Platelet count	× 10^∧^9/L
Red cell count	× 10^∧^12/L
Packed cell volume	L/L
Mean corpuscular volume	fL
Mean corpuscular hemoglobin	pg
Mean corpuscular hemoglobin concentration	g/L
Red cell distribution width	%
Mean platelet volume	fL
Neutrophils	× 10^∧^9/L & %
Lymphocytes	× 10^∧^9/L & %
Monocytes	× 10^∧^9/L & %
Eosinophils	× 10^∧^9/L & %
Basophils	× 10^∧^9/L & %
*Biochemical Analyses*	
Sodium	mmol/L
Chloride	mmol/L
Bicarbonate	mmol/L
Creatinine	umol/L
Urea	mmol/L
Anion gap	mmol/L
Calcium	mmol/L
Phosphate	mmol/L
Albumin	g/L
Total protein	g/L
Bilirubin	umol/L
Globulin	g/L
Alkaline phosphatase	U/L
Alanine aminotransferase	U/L
Aspartase aminotransferase	U/L
Gamma Glutamyl Transpeptidase	U/L
Lactate dehydrogenase	U/L
Urate	mmol/L
Estimated glomerular filtration rate	mL/min/1.73 m^∧^2
Ionized calcium calculated	mmol/L

#### Physical Examination

The nurse practitioner undertakes a physical examination of the patients' lungs and heart sounds, checks for peripheral oedema, and performs any other examinations deemed clinically relevant for the individual.

## Discussion

This clinic is the first example of a standard care Australian, nurse practitioner-led, postpartum intervention clinic and registry for women who experience serious pregnancy complications associated with an increased risk of future cardiovascular risk. This new model of care is an evolution of cardiac rehabilitation and prevention strategies and resources, usually reserved for secondary prevention of ischemic heart disease, into the primordial and primary prevention arena.

Awareness of the relationship between pregnancy complications and CVD remains poor in both the medical community and general population, despite wide acceptance of the more traditional risk factors for heart disease. Improving this awareness through widespread community education will likely have a profound impact on the uptake of postpartum health assessments, both by patients and primary care providers. Future research stemming from our model of care will aim to engage with and educate patients, general community members, primary care physicians, and antenatal care teams, and to drive guideline writing in Australia to ensure follow-up protocols are consistent and clear. Pinpointing the ideal time to intervene and educate women following pregnancy complications will also be a main future priority.

The nurse practitioner leading this postpartum intervention clinic provides a holistic approach to cardiovascular and overall health. Family participation is also encouraged, where possible, through the promotion of healthy eating habits and physical activity practices that can include partners and children. This clinic therefore holds potential to improve health for the whole family, although this will not be quantitatively assessed.

The described postpartum clinic services the northern Adelaide area, which is complicated by some of the highest rates of chronic disease, smoking, obesity and physical inactivity, diabetes, heart disease, mental illness, and socioeconomic disadvantage in urban Australia (49). The aim of our preventive clinic in an area burdened by this level of disease and social disadvantage is to prevent these women from progressing to serious cardiovascular and metabolic disease. Such early intervention may help alleviate some of the significant burden of disease in the northern Adelaide area.

The quantitative success of this postpartum intervention clinic approach will be evaluated on an ongoing basis through comparison of baseline results with data from future appointments. Future directions will explore development and validation of cardiovascular risk algorithms specific to younger women, as current risk scores are not suitable for this population. Future research will also focus on developing strategies for earlier identification of women who are less likely to engage with the health system following birth of their baby. This will inform particular social factors that identify these high-risk individuals and provide potential opportunities to develop interventions that are more effective.

## Data Availability Statement

The datasets generated for this study will not be made publicly available as the authors are not permitted to share datasets for this research.

## Ethics Statement

The studies involving human participants were reviewed and approved by Central Adelaide Local Health Network Human Research Ethics Committee. Written informed consent for participation was not required for this study in accordance with the national legislation and the institutional requirements.

## Author Contributions

EA prepared the manuscript. All authors were involved in the design of the clinic and associated research study. All authors edited the draft manuscript, provided critical feedback, and approved the final manuscript.

### Conflict of Interest

The authors declare that the research was conducted in the absence of any commercial or financial relationships that could be construed as a potential conflict of interest.
